# Supplementary material for: Mettl3-mediated mRNA m6A modification controls postnatal liver development by modulating the transcription factor Hnf4a
Source: Nat Commun. 2022 Aug 5;13:4555. doi: 10.1038/s41467-022-32169-4 (PMC9355946; doi:10.1038/s41467-022-32169-4)
Supplement: Supplementary file 1 — Supplementary Information [file 41467_2022_32169_MOESM1_ESM.pdf]

Mettl3-mediated mRNA m^{6}A modification controls postnatal liver development by modulating the transcription factor Hnf4a

Yan Xu[1, 3, *], Zhuowei Zhou[1, *], Xinmei Kang[1, *], Lijie Pan[1, 2], Chang Liu[1], Xiaoqi Liang[1, 2], Jiajie Chu[1, 2], Shuai Dong[1], Yanli Li[1], Qiuli Liu[1], Yuetong Sun[1], Shanshan Yu[1, 2], Qi Zhang[1, 2, 3, #]

Contents	Pages
Supplementary Figures and Figure legends	2 to 19
Supplementary Tables	20 to 28
Supplementary Datasets	29
Supplementary References	30

Supplementary materials: 9 Figures; 3 Tables; 5 Datasets

Supplementary Figures and Figure legends

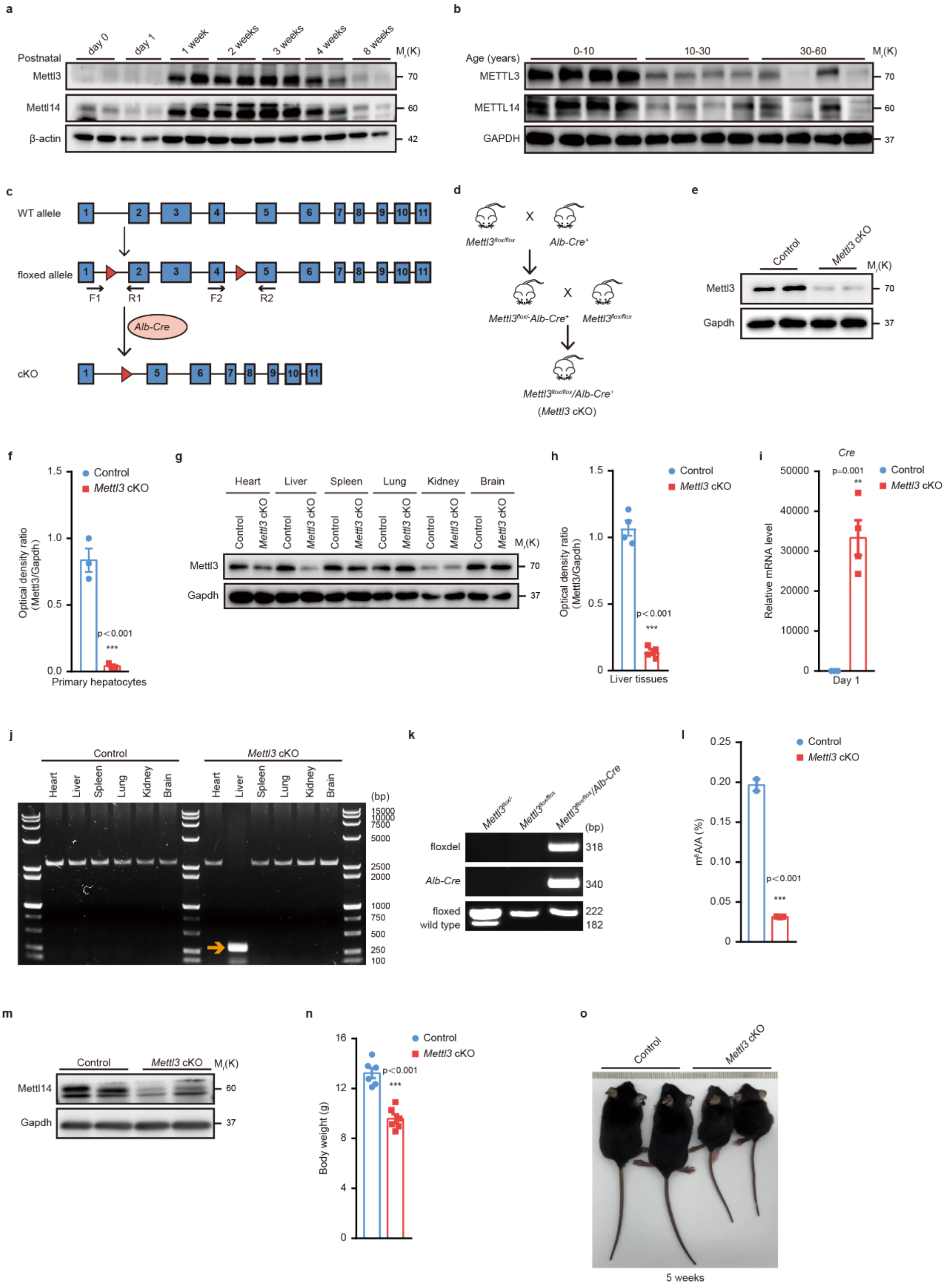

Supplementary Fig. 1. Liver-specific Mettl3 knockout mouse construction and characterization.

(a) Western blot for Mettl3 and Mettl14 of mouse liver tissues at indicated time points

after birth (3 experiments were repeated independently with similar results). (b) Western blot for METTL3 and METTL14 of human liver tissues at indicated ages (3 experiments were repeated independently with similar results). (c) Targeting strategy of *Albumin*-enhancer/promoter-driven *Cre*-mediated *Mettl3* conditional knockout mouse construction. Floxed-*Mettl3* allele was generated by flanking exons 2 and 4 with loxP sites. Hepatocyte-specific conditional knockout of Mettl3 (*Mettl3* cKO) was achieved by crossing floxed-*Mettl3* mice with *Alb*-enhancer/promoter-driven *Cre* transgenic mice. (d) Schematic diagram of breeding strategies of *Mettl3* cKO mice. (e) Western blot for Mettl3 in hepatocytes isolated from Control and *Mettl3* cKO mice (3 experiments were repeated independently with similar results). (f) Densitometry analysis of western blot for Mettl3 in hepatocytes isolated from Control and *Mettl3* cKO mouse livers in (e) (n = 3 for each group over 3 independent experiments). (g) Western blot for Mettl3 in indicated tissues from Control and *Mettl3* cKO mice (3 experiments were repeated independently with similar results). (h) Densitometry analysis of western blot for Mettl3 in Control and *Mettl3* cKO liver tissues in (g) (n = 4 for each group). (i) Quantitation of *Cre* mRNA expression of Control and *Mettl3* cKO liver tissue at day 1 after birth (n = 3 Control group; n = 4 for cKO group). (j) Genomic PCR analysis for floxdel allele (amplified with *Mettl3*-F1 and *Mettl3*-R2 primer shown on (c) in indicated tissues from Control and *Mettl3* cKO mice (3 experiments were repeated independently with similar results). (k) Genomic PCR analysis of 1-day-old mouse of indicated genotype (3 experiments were repeated independently with similar results), showing successful knockout of *Mettl3* at day 1

after birth in *Mettl3* cKO mouse liver. Floxdel fragments were detected using liver samples as templates, and *Alb*-*Cre* and floxed/wild type fragments were detected using mouse tail samples as templates. (l) LC-MS/MS analysis of m^6A mRNA modification in Control and *Mettl3* cKO mouse liver tissue (n = 2 for Control group; n = 3 for cKO group). (m) Western blot for Mettl14 of Control and *Mettl3* cKO mouse liver tissues 2 weeks after birth (3 experiments were repeated independently with similar results). (n) Body weight of female Control and *Mettl3* cKO littermates at 4 weeks after birth (n = 6 for each group). (o) Representative appearances of Control and *Mettl3* cKO littermates at 5 weeks after birth (10 experiments were repeated independently with similar results). Data in (f), (h), (i), (l), and (n) were shown as mean ± SEM with the indicated significance (***P < 0.001; two-tailed student's *t*-test). Source data are provided as a Source Data file.

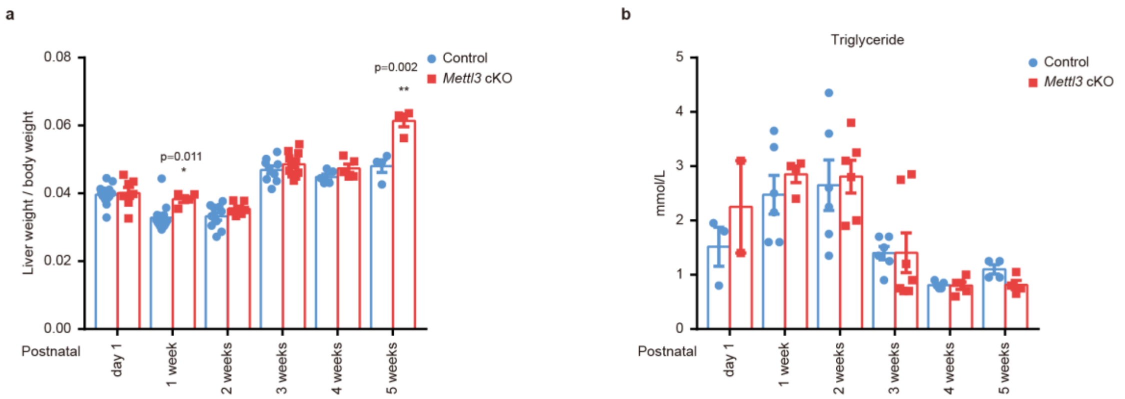

Supplementary Fig. 2. Liver weight to body weight ratio and serum triglyceride levels of Control and *Mettl3* cKO mice.

(a) Ratio of liver weight to body weight of Control and *Mettl3* cKO mice at different time points postnatally (n = 4 for 1 week cKO group and 5 weeks groups; n = 5 for 4 weeks groups; n = 6 for 2 weeks cKO group; n = 7 for day 1 cKO group; n = 9 for 3 weeks Control group; n = 12 for 3 weeks cKO group; n = 15 for day 1 Control group and 1 week Control group). (b) Serum levels of triglyceride of Control and *Mettl3* cKO mice at different time points postnatally (n = 2 for day 1 cKO group; n = 3 for day 1 Control group; n = 4 for 1 week cKO group; n = 5 for 4 weeks groups; n = 7 for 3 weeks cKO group; n = 6 for other groups). Data in (a) and (b) were shown as mean ± SEM with the indicated significance (*p < 0.05, **p < 0.01; two-tailed student's *t*-test). Source data are provided as a Source Data file.

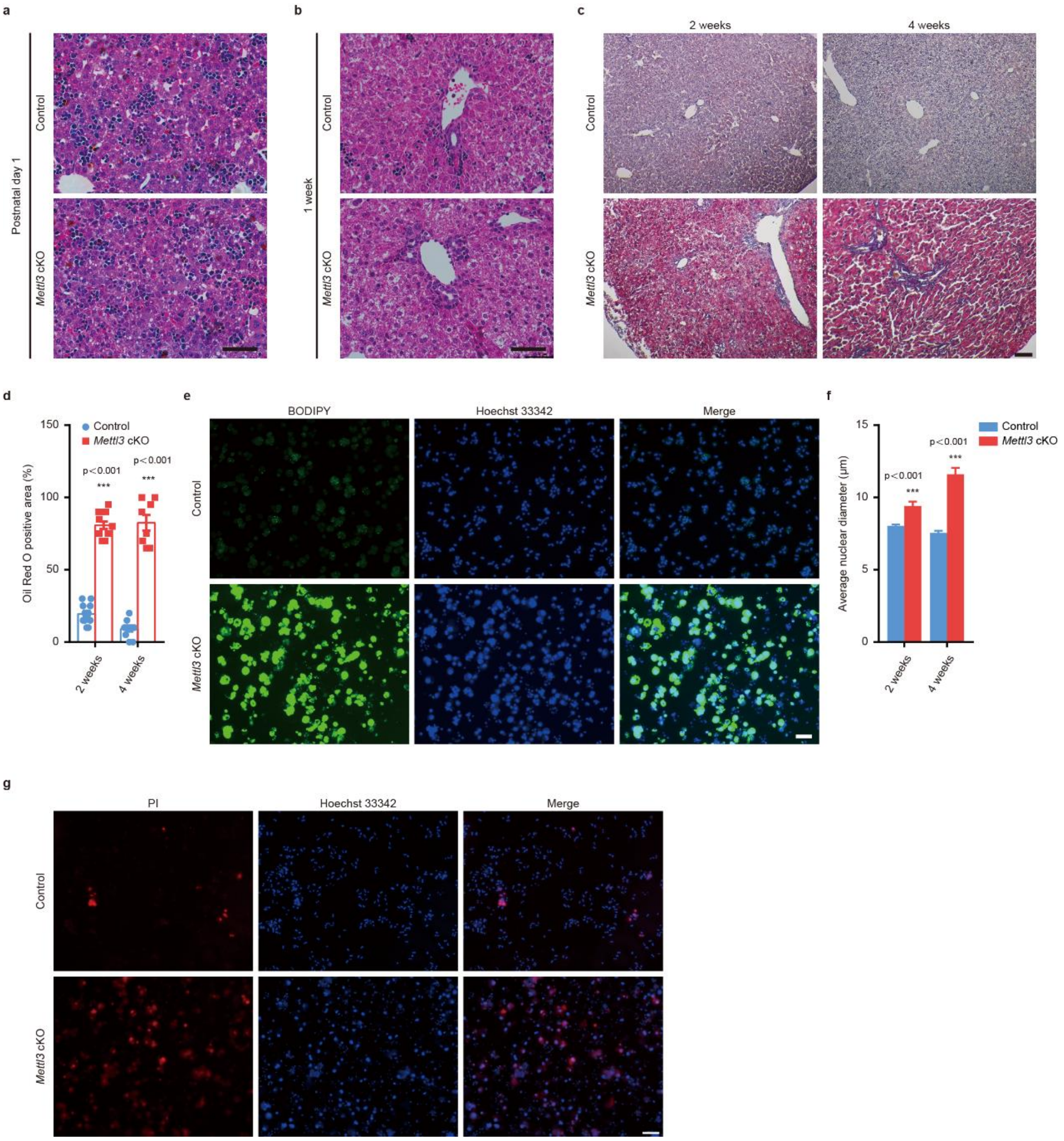

Supplementary Fig. 3. Hepatic Mettl3 deletion results in liver injury.

(a) and (b) Representative H&E staining photographs of liver sections from Control and *Mettl3* cKO mice at 1 day (a) and 1 week (b) after birth (10 experiments were repeated independently with similar results). Scale bar = 50 μm. (c) Representative Oil Red O staining photographs of liver sections from Control and *Mettl3* cKO mice at 2 weeks and 4 weeks postnatally (6 experiments were repeated independently with similar results). Scale bar = 100 μm. (d) Quantification of Oil Red O positive area shown in (c) (n = 8 for 4 weeks cKO group; n = 12 for other groups). (e)

Representative BODIPY staining fluorescent photographs of primary hepatocytes isolated from Control and *Mettl3* cKO mice (6 experiments were repeated independently with similar results). Scale bar = 100 μm. (f) Quantitation of average nuclear diameter of liver sections from Control and *Mettl3* cKO mice (n = 45 for 4 weeks cKO group; n = 56 for 2 weeks cKO group; n = 67 for 2 weeks Control group; n = 78 for 4 weeks Control group). (g) Representative PI staining photographs of primary hepatocytes isolated from Control and *Mettl3* cKO mice (3 experiments were repeated independently with similar results). Scale bar = 100 μm. Data in (d) and (f) were shown as mean ± SEM with the indicated significance (**p < 0.01, ***p < 0.001; two-tailed student's *t*-test). Source data are provided as a Source Data file.

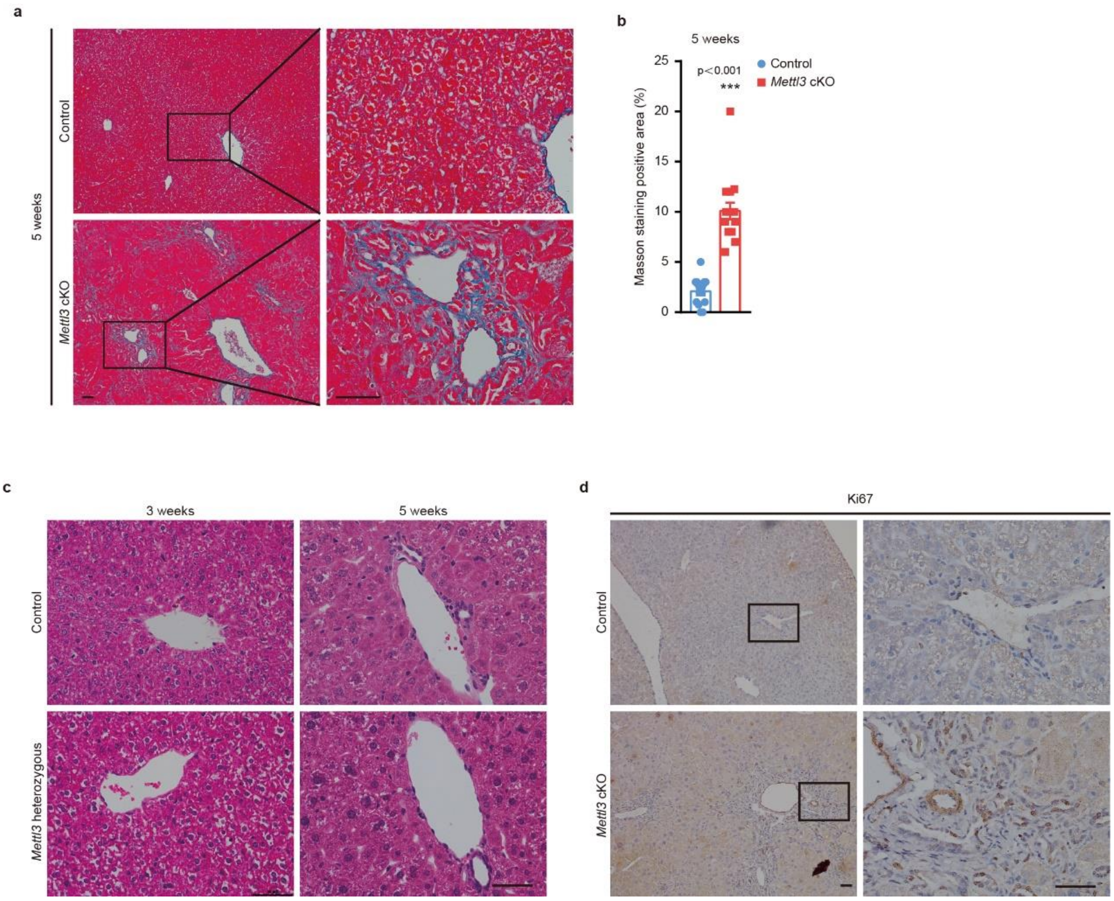

Supplementary Fig. 4. Histological analysis of Control and *Mettl3* cKO liver sections.

(a) Representative Masson's trichrome staining photographs of liver sections from Control and *Mettl3* cKO mice at 5 weeks after birth. Scale bar = 50 μm. (b) Quantification of Masson's trichrome staining positive area (n = 15 for each group). (c) Representative H&E staining photographs of liver sections from Control and *Mettl3* heterozygous mice at 3 weeks and 5 weeks after birth (10 experiments were repeated independently with similar results). Scale bar = 50 μm. (d) Representative Ki67 immunohistochemistry staining photographs of liver sections from 5-week-old Control and *Mettl3* cKO mice (6 experiments were repeated independently with similar results). Scale bar = 50 μm. Data in (b) were shown as mean ± SEM with the

indicated significance (***$p < 0.001$; two-tailed student's *t*-test). Source data are provided as a Source Data file.

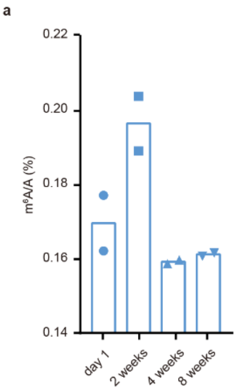

b

Sample	Peak number	Annotated gene number	Number of annotated genes consistent on replicates
day 1	15139	6330	4294
1 week	12483	5522	3155
2 weeks	12615	5515	1604
4 weeks	10561	4856	2468
8 weeks	6806	3445	780

c

	Emerging peaks	Resolving peaks
1 week/day 1	760	1440
2 weeks/1 week	1097	1075
4 weeks/2 weeks	579	1724
8 weeks/4 weeks	555	2357

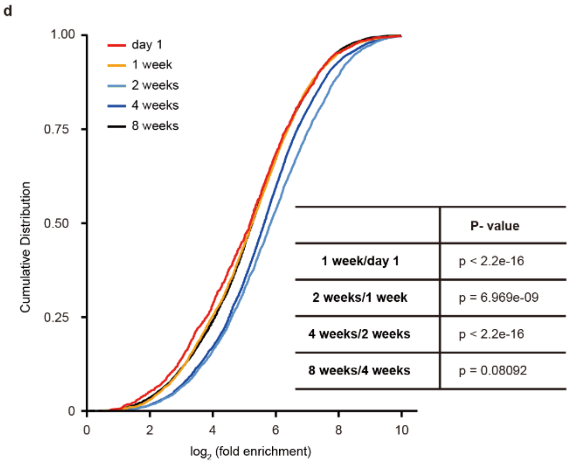

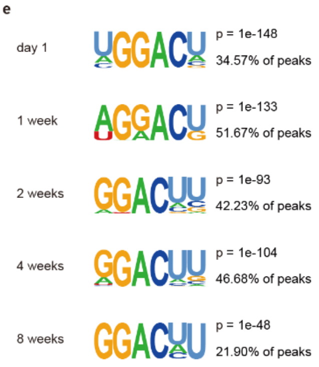

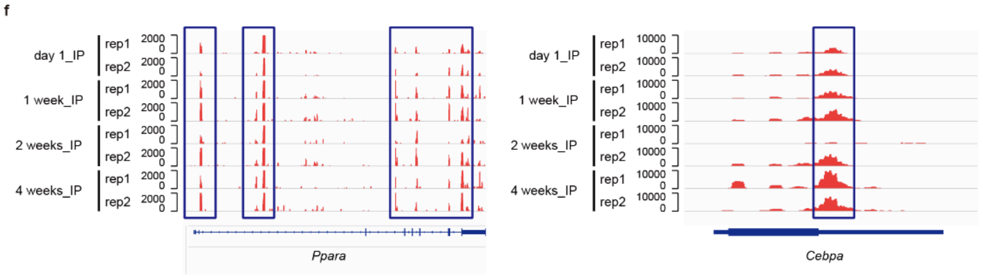

Supplementary Fig. 5. Dynamic m^{6}A modifications in postnatal mouse liver development.

(a) LC-MS/MS analysis of m^{6}A modification in mouse liver tissues collected at indicated time points (n = 2 for each group). (b) Summary of peak number, the number of annotated genes, and the number of annotated genes in each group in m^{6}A-RIP sequencing. (c) Summary of dynamic changes of m^{6}A-immunoprecipitated peaks in different stages of postnatal liver development. (d) Cumulative distribution

of m^6A-immunoprecipitated peak enrichment in mouse liver tissue at indicated time points. Kolmogorov-Smirnov test (KS-test) was performed to compare m^6A distributions among indicated ages. (e) Motif analysis of the identified m^6A-immunoprecipitated peaks. P-value was calculated automatically by HOMER software. (f) Genome Browser screenshots of m^6A-RIP sequencing read density signals under 4 different time points after birth at *Ppara* and *Cebpa* locus. Two replicates were contained for each time point. Source data are provided as a Source Data file.

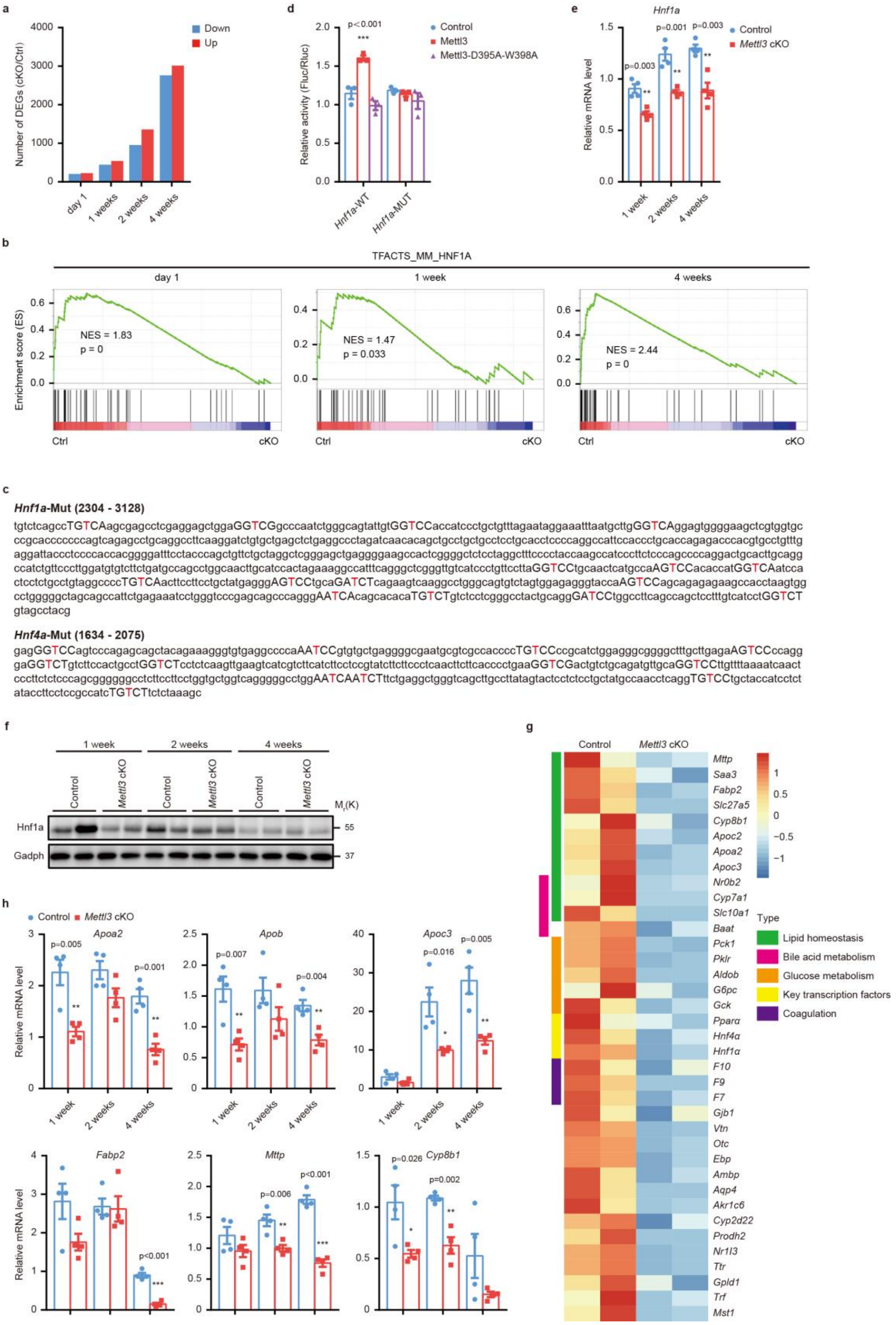

Supplementary Fig. 6. Mettl3-mediated m^{6}A regulates the liver-enriched transcription factor network.

(a) Numbers of differentially regulated genes (DEGs) of Control and *Mettl3* cKO mice at indicated time points after birth. (b) Gene set enrichment analysis (GSEA) for Hnf1a pathways in RNA-sequencing data of Control and *Mettl3* cKO liver tissues at 1 day, 1 week, and 4 weeks after birth. (c) WT and mutated (Mut) m^6A-enriched regions of *Hnf1a* and *Hnf4a* were used in dual-luciferase reporter assays. Putative m^6A consensuses were in bold while the mutation sites in red. (d) Dual-luciferase reporter assays showing the effect of WT and catalytic mutant Mettl3 on *Hnf1a* reporters with either WT or mutated m^6A-modified sites (n = 3 for each group over 3 independent experiments). (e) RT-qPCR for mRNA levels of *Hnf1a* at different time points in Control and *Mettl3* cKO mouse liver tissues (n = 4 for each group). (f) Western blot for Hnf1a from Control and *Mettl3* cKO mice liver tissues at 1 week, 2 weeks, and 4 weeks after birth (3 experiments were repeated independently with similar results). (g) The heatmap showing expression levels of Hnf4a downstream targets in 4-week-old Control and *Mettl3* cKO mouse livers in RNA-sequencing data. (h) RT-qPCR analysis of Hnf4a target genes from Control and *Mettl3* cKO mouse liver tissues at different postnatal time points (n = 4 for each group). Data in (d), (e), and (h) were shown as mean ± SEM with the indicated significance (*p<0.05, **p < 0.01, ***p < 0.001; two-tailed student's *t*-test). Source data are provided as a Source Data file.

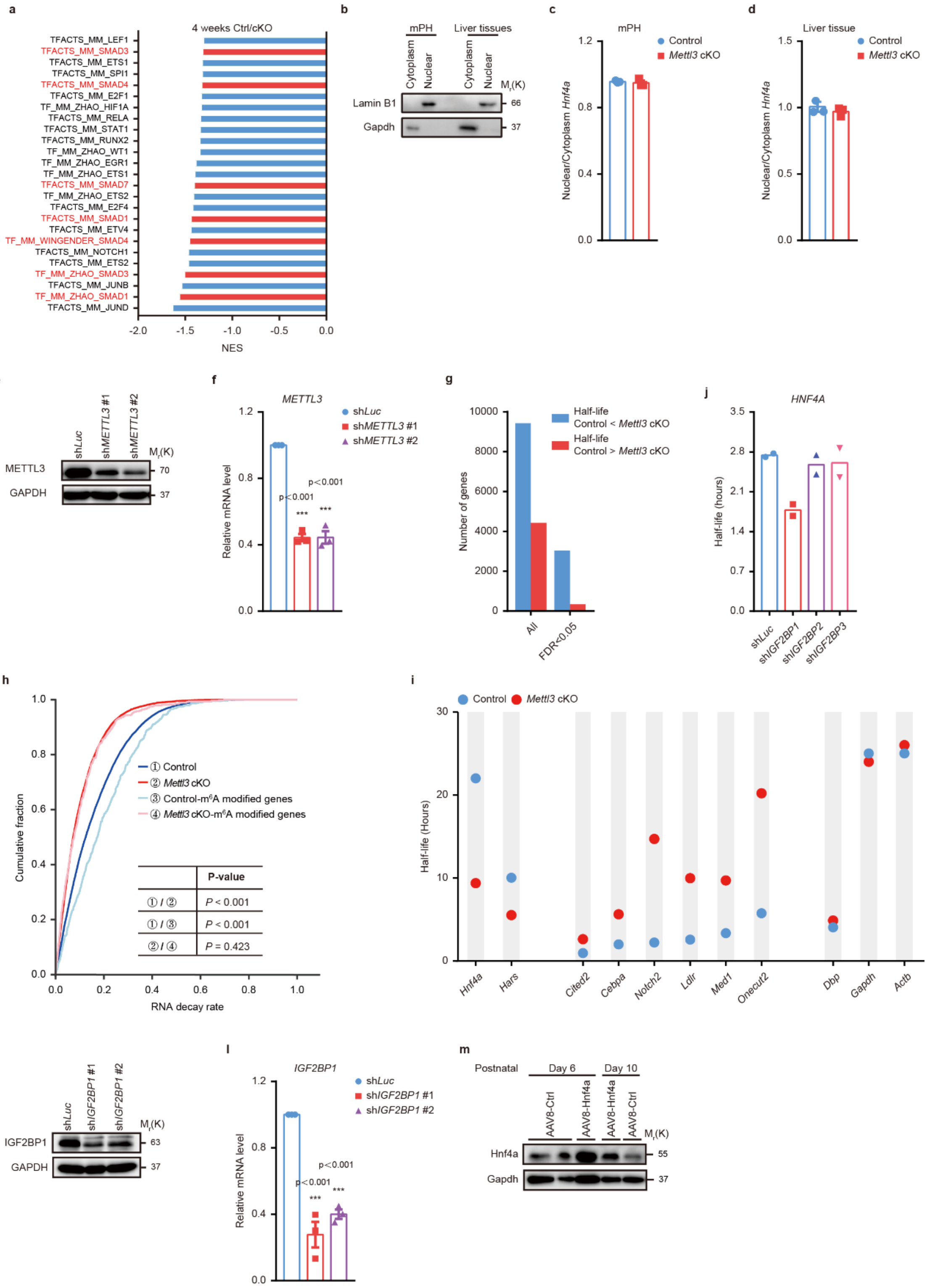

Supplementary Fig. 7. Mettl3 knockout decreases *Hnf4a* mRNA stability in postnatal liver development.

(a) GSEA analysis of RNA-sequencing showed that *Smad* signaling was significantly enriched in *Mettl3* cKO liver tissues at 4 weeks after birth. (b) Western blot showing

Lamin B1 (a marker for nuclear fraction) and Gapdh (marker for cytoplasm fraction) protein level in isolated cytoplasm and nuclear fractions from mouse primary hepatocytes and liver tissues (3 experiments were repeated independently with similar results), indicating successful separation of two fractions. (c) Nuclear/cytoplasm *Hnf4a* mRNA level in Control and *Mettl3* cKO primary hepatocytes (n = 3 for each group). (d) Nuclear/cytoplasm *Hnf4a* mRNA level in Control and *Mettl3* cKO liver tissues (n = 3 for each group). (e) Western blot of METTL3 in HepG2 cells with METTL3 knockdown with 2 independent small Hairpin RNA (shRNA) targeting *METTL3*. shRNA targeting firefly luciferase (sh*Luc*) was used as a control (also hereafter in similar experiments). (f) *METTL3* relative mRNA level in HepG2 cells with or without METTL3 knockdown (n = 3 for each group). (g) Histogram showing differences of mRNA half-life between Control and *Mettl3* cKO primary hepatocytes. (h) Cumulative fraction curves of total or m^6A-modified genes of primary hepatocytes from Control and *Mettl3* cKO mice showing differential global RNA decay rate in each group. (i) Half-life of selected genes with downregulated, upregulated, or unchanged mRNA stability between Control and *Mettl3* cKO primary hepatocytes. Data in (g), (h), and (i) were analyzed from RNA-sequencing data in Supplementary Dataset 5. (j) *HNF4A* mRNA half-life in IGF2BP1, IGF2BP2, or IGF2BP3 knockdown in HepG2 cell line. Results were collected from published RNA-sequencing data (GSE90684). (k) Western blot of IGF2BP1 in HepG2 cells with or without IGF2BP1 knockdown. (l) *IGF2BP1* relative mRNA level in HepG2 cells with or without IGF2BP1 knockdown (n = 3 for each group). (m) *Mettl3* cKO mice

were intravenously injected with AAV8-Ctrl or AAV8-Hnf4a at day 2, and liver tissues were collected at indicated time points for Western blot of Hnf4a (3 experiments were repeated independently with similar results). Data in (c), (d), (f), (j) and (l) were shown as mean ± SEM with the indicated significance (***$p < 0.001$; two-tailed student's *t*-test). Source data are provided as a Source Data file.

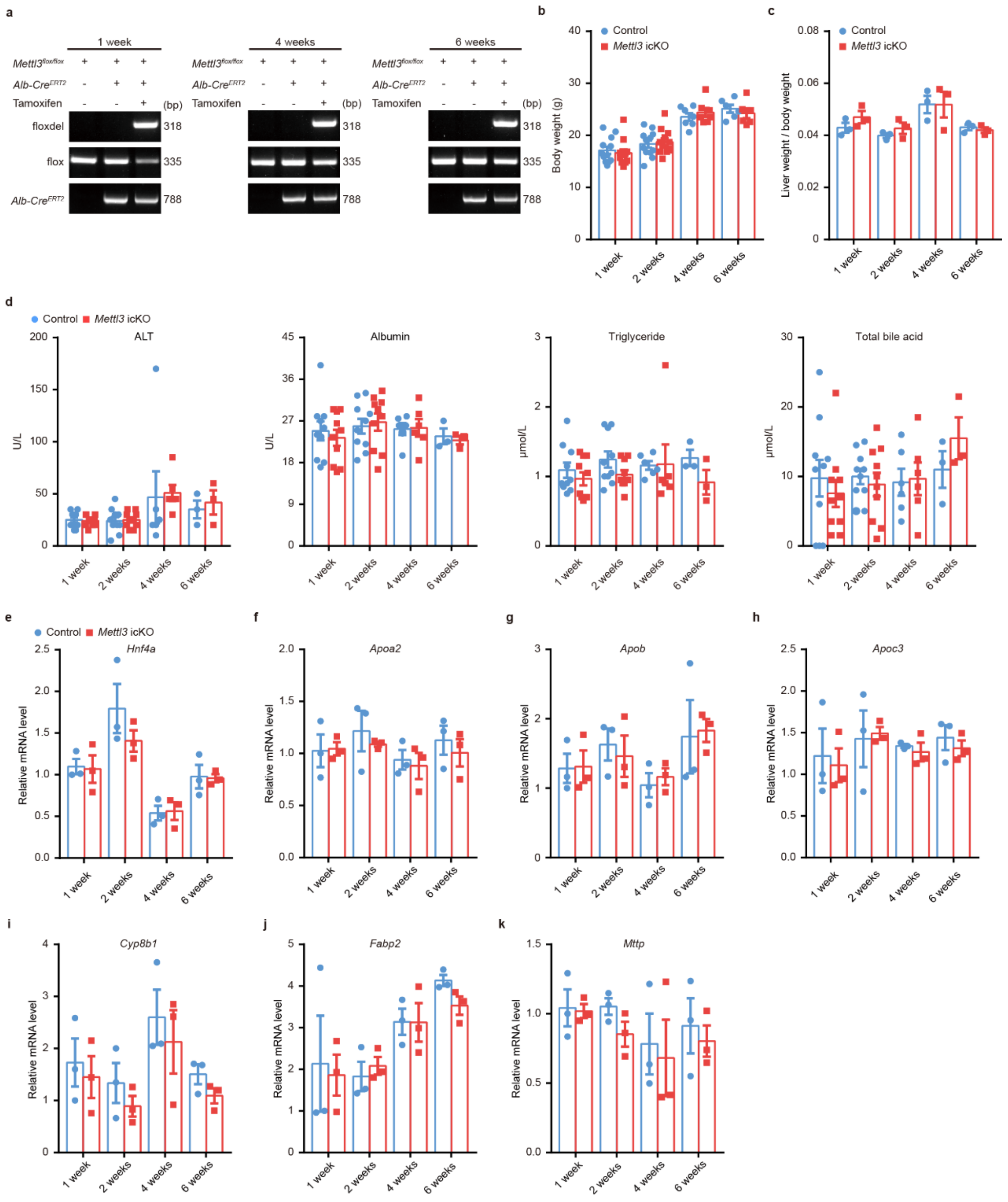

Supplementary Fig. 8. Mettl3 has minimal effects on adult liver homeostasis.

(a) Genomic PCR of Control and inducible liver-specific *Mettl3* knockout (*Mettl3* icKO) mice (3 experiments were repeated independently with similar results). Floxdel fragments were detected using liver samples as templates by *Mettl3*-F1 and *Mettl3*-R2 primers. Flox and *Alb-Cre*^{ERT} fragments were detected using mouse tail samples as templates. Flox fragments were amplified by *Mettl3*-F2 and *Mettl3*-R2 primers, and

Alb-*Cre*^{ERT} fragments were amplified by *Alb*-*CreERT*-F and *Alb*-*CreERT*-R primers. (b) Body weight of Control and *Mettl3* icKO littermates at different time points after tamoxifen treatment (n = 6 for 6 weeks groups; n = 7 for 4 weeks groups; n = 12 for other groups). (c) The ratio of liver weight to body weight of Control and *Mettl3* icKO mice at different time points after tamoxifen treatment (n = 3 for each group). (d) Serum levels of AST, ALB, Triglyceride, and Total bile acid of Control and *Mettl3* icKO mice at different time points after tamoxifen treatment (n = 3 for 6 weeks groups; n = 6 for 4 weeks groups; n = 10 for other groups). (e-k) RT-qPCR analysis of *Hnf4a* (e) and Hnf4a downstream targets *Apoa2* (f), *Apob* (g), *Apoc3* (h), *Cyp8b1* (i), *Fabp2* (j), and *Mttp* (k) for Control and *Mettl3* icKO mouse liver tissues at different time points after tamoxifen treatment (n = 3 for each group). Data in (b)-(k) were shown as mean ± SEM. Source data are provided as a Source Data file.

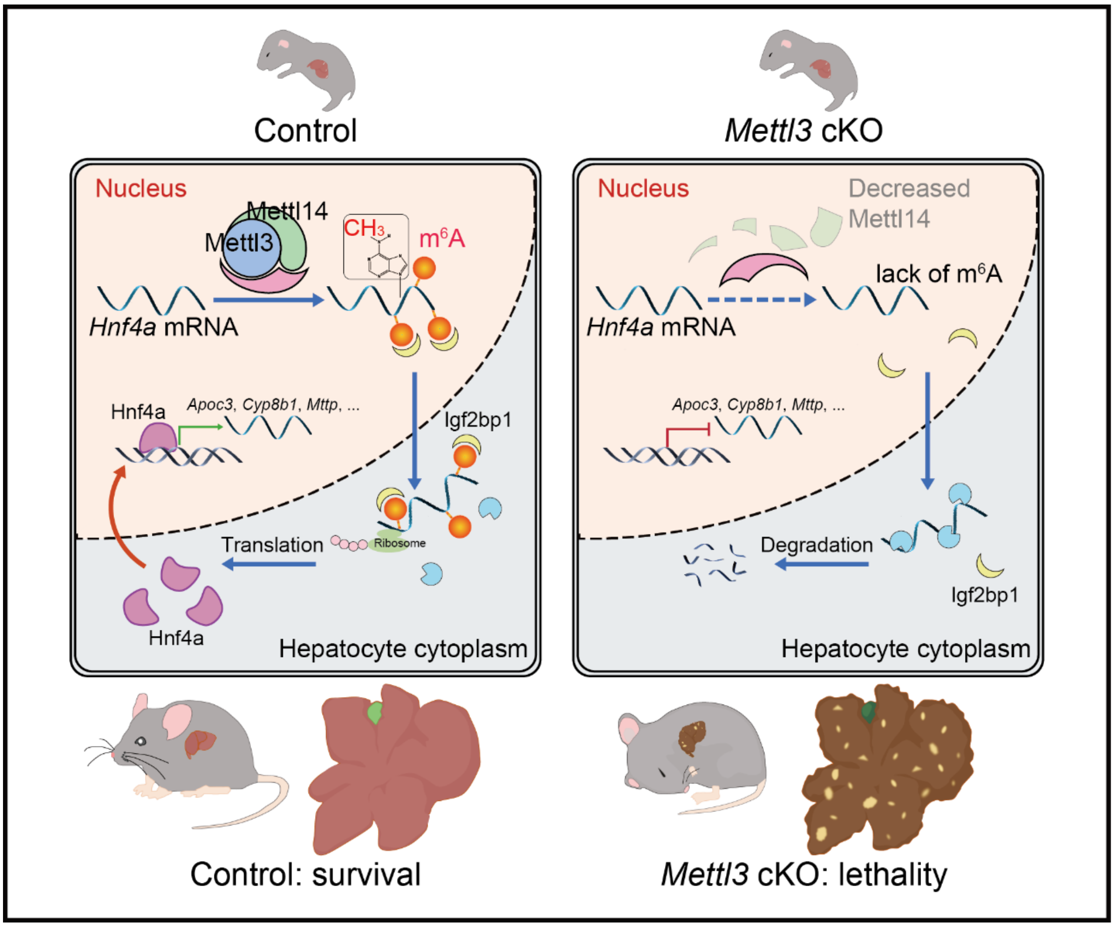

Supplementary Fig. 9. Graphic abstract showing Mettl3-mediated m^{6}A modification controls postnatal liver development by modulating the transcription factor Hnf4a.

Supplementary Tables

Supplementary Table 1. Primers

1. Primers for molecular cloning and genotyping

Name	Sequences
Mettl3-F1	5'-AGAGGAGGAGAAGGTGGCAGAG-3'
Mettl3-R1	5'-CCTTTCATTCACATGGCAGCAC-3'
Mettl3-F2	5'-AGGCTCACTTGCAAGTAAAAGGAAG-3'
Mettl3-R2	5'-AGGCCTATAATCCTAGCACTG-3'
Alb-Cre-F	5'-GGGCAGTCTGGTACTTCCAAGCT-3'
Alb-Cre-R	5'-TAGCTACCTATGCGATCCAAACAAC-3'
Alb-CreERT-F	5'-TCTCCCCACCTCTAGCCCAAAGAAA-3'
Alb-CreERT-R	5'-AGGCAGAGGACTGTATTGATCAGTC-3'
Mettl3-PKD-F	5'-CCAGGCCTAAGCTTACGCGTATGTCGGACACGTGGAGCTC-3'
Mettl3-PKD-R	5'-TCGTCCTTGTAGTCGAATTCTAAATTCTTAGGTTTAGAGA-3'
Mettl3-AWWA-F	5'-TGTGATGGCTGCCCCACCTGCGGATATTCACATGGAGCTA-3'
Mettl3-AWWA-R	5'-TGTGAATATCCGCAGGTGGGGCAGCCATCACAACTGCAAA-3'

2. Primers for TRC lentiviral vectors encoding shRNA construction

Name	Sequences
sh*IGF2BP1*-1-F	5'-CCGGGCAGTGGTGAATGTCACCTATCTCGAGATAGGTGACATTCACCACTGCTTTTTG-3'
sh*IGF2BP1*-1-R	5'-AATTCAAAAAGCAGTGGTGAATGTCACCTATCTCGAG

	ATAGGTGACATTCACCACTGC-3'
sh*IGF2BP1*-2-F	5'-CCGGCTCCGCTTGTAAGATGATCTTCTCGAGAAGATC ATCTTACAAGCGGAGTTTTTG-3'
sh*IGF2BP1*-2-R	5'-AATTCAAAAACTCCGCTTGTAAGATGATCTTCTCGAG AAGATCATCTTACAAGCGGAG-3'
sh*METTL3*-1-F	5'-CCGGCGTCAGTATCTTGGGCAAGTTCTCGAGAACTTG CCCAAGATACTGACGTTTTTG-3'
sh*METTL3*-1-R	5'-AATTCAAAAA CGTCAGTATCTTGGGCAAGTT CTCGAG AACTTGCCCAAGATACTGACG-3'
sh*METTL3*-2-F	5'-CCGGGCCAAGGAACAATCCATTGTTCTCGAGAACAAT GGATTGTTCCTTGGCTTTTTG-3'
sh*METTL3*-2-R	5'-AATTCAAAAA GCCAAGGAACAATCCATTGTT CTCGAG AACAATGGATTGTTCCTTGGC-3'

3. Primers for RT-qPCR

**Name**	**Sequences**
Cre-RT-F	5'-CTCTGACAGATGCCAGGACA-3'
Cre-RT-R	5'-GATCAGCATTCTCCCACCAT-3'
human *GAPDH*-RT-F	5'-AATGAAGGGGTCATTGATGG-3'
human *GAPDH*-RT-R	5'-AAGGTGAAGGTCGGAGTCAA-3'
human *IGF2BP1*-RT-F	5'-GGCCATCGAGAATTGTTGCAG-3'
human *IGF2BP1*-RT-R	5'-CCAGGGATCAGGTGAGACTG-3'
human *HNF4A*-RT-F	5'-TGCGACTCTCCAAAACCCTC-3'
human *HNF4A*-RT-R	5'-ATTGCCCATCGTCAACACCT-3'
human *METTL3*-RT-F	5'-GAAGCAGCTGGACTCTCTGC-3'
human *METTL3*-RT-R	5'-ACGGAAGGTTGGAGACAATG-3'
mouse *Acta2*-RT-F	5'-CTGACAGAGGCACCACTGAA-3'
mouse *Acta2*-RT-R	5'-CATCTCCAGAGTCCAGCACA-3'

mouse *AFP*-RT-F	5'-ATGAAACCTATGCCCCTCCC-3'
mouse *AFP*-RT-R	5'-TCAGGCTTTTGCTTCACCAGG-3'
mouse *Albumin*-RT-F	5'-CATGACACCATGCCTGCTGAT-3'
mouse *Albumin* -RT-R	5'-CTCTGATCTTCAGGAAGTGTAC-3'
mouse *Apoa2*-RT-F	5'-CTCCTGCGGCTAAGTGAGATG-3'
mouse *Apoa2*-RT-R	5'-GAAAACAGGCAGAAGGTAGGGA-3'
mouse *Apob*-RT-F	5'-TACCTTGGGCATCGACACAC-3'
mouse *Apob*-RT-R	5'-GTGCTGATGCTGCTCTCGTA-3'
mouse *Apoc3*-RT-F	5'-GAACAAGCCTCCAAGACGGT -3'
mouse *Apoc3*-RT-R	5'-GTTGGTTGGTCCTCAGGGTT-3'
mouse *Col1a1*-RT-F	5'-TGACTGGAAGAGCGGAGAGT-3'
mouse *Col1a1*-RT-R	5'-GTTCGGGCTGATGTACCAGT-3'
mouse *Cyp8b1*-RT-F	5'-GGTACGCTTCCTCTATCGCC-3'
mouse *Cyp8b1*-RT-R	5'-GGATGGCGTCTTATGGGGAG-3'
mouse *Epcam*-RT-F	5'-GGAGTCCCTGTTCCATTCTTCT-3'
mouse *Epcam*-RT-R	5'-GCGATGACTGCTAATGACACCA-3'
mouse *Fabp2*-RT-F	5'-TGGAAAGGAGCTGATTGCTGT-3'
mouse *Fabp2*-RT-R	5'-AAGAATCGCTTGGCCTCAACT-3'
mouse *Gapdh*-RT-F	5'-CATGGCCTTCCGTGTTCCT-3'
mouse *Gapdh*-RT-R	5'-GCCTGCTTCACCACCTTCT-3'
mouse *Hnf1a*-RT-F	5'-ACTTGCAGCAGCACAACATC-3'
mouse *Hnf1a*-RT-R	5'-GAATTGCTGAGCCACCTCTC-3'
mouse *Hnf4a*-RT-F	5'-AGAGGTTCTGTCCCAGCAGA-3'
mouse *Hnf4a*-RT-R	5'-ATGTACTTGGCCCACTCGAC-3'
mouse *Igf2bp1*-RT-F	5'-CGGCAACCTCAACGAGAGT-3'
mouse *Igf2bp1*-RT-R	5'-GTAGCCGGATTTGACCAAGAA-3'
mouse *Krt7*-RT-F	5'-AGGAGATCAACCGACGCAC-3'
mouse *Krt7*-RT-R	5'-CACCTTGTTCGTGTAGGCG-3'
mouse *Krt19*-RT-F	5'-GTCCTACAGATTGACAATGC-3'

mouse *Krt19*-RT-R	5'-CACGCTCTGGATCTGTGACAG-3'
mouse *Mettl3*-RT-F	5'-CTGGGCACTTGGATTTAAGGAA-3'
mouse *Mettl3*-RT-R	5'-TGAGAGGTGGTGTAGCAACTT-3'
mouse *Mttp*-RT-F	5'-CCAGGGCTTTTGCCTTGAAC-3'
mouse *Mttp*-RT-R	5'-GAGGACCTGTCCCACAATGG-3'
mouse *Pdgfrb*-RT-F	5'-TTCCAGGAGTGATACCAGCTT-3'
mouse *Pdgfrb* -RT-R	5'-AGGGGGCGTGATGACTAGG-3'
mouse *Sox9*-RT-F	5'-AGTACCCGCATCTGCACAAC-3'
mouse *Sox9*-RT-R	5'-ACGAAGGGTCTCTTCTCGCT-3'

4. Primers for m^{6}A-RIP-qPCR

**Name**	**Sequences**
mouse *Apof*-positive-m^{6}A-1-RT-F	5'-GCCAAGGAACGAGGTCGAGA-3'
mouse *Apof*-positive-m^{6}A-1-RT-R	5'-AAGAGCAGCCGTGATCACCG-3'
mouse *Apof*-negative-m^{6}A-RT-F	5'-GCCCCCTGTCTCCAAATTCC-3'
mouse *Apof*-negative-m^{6}A-RT-R	5'-AAGCTGAAGCTGAAGAGCCC-3'
mouse *Cebpa*-positive-m^{6}A-1-RT-F	5'-GGCTGGAGACCCAGAGGATG-3'
mouse *Cebpa*-positive-m^{6}A-1-RT-R	5'-CTTTCAGGCCACACCGGAAT-3'
mouse *Cebpa*-negative-m^{6}A-RT-F	5'-CCGCCTTCAACGACGAGTTC-3'
mouse *Cebpa*-negative-m^{6}A-RT-R	5'-CCGGGTAGTCAAAGTCACCG-3'
mouse *Cited2*-positive-m^{6}A-1-RT-F	5'-ATCAAGGAGCTGCCCGAACT-3'
mouse *Cited2*-positive-m^{6}A-1-RT-R	5'-CAACAGCTGACTCTGCTGGG-3'
mouse *Cited2*-negative-m^{6}A-RT-F	5'-ATGATGGCCATGAACCACGG-3'
mouse *Cited2*-negative-m^{6}A-RT-R	5'-CTGCTGCTGGTGATGATGCG-3'
mouse *Egfr*-positive-m^{6}A-1-RT-F	5'-CTTCTGGTAGCCATGCCCAC-3'
mouse *Egfr*-positive-m^{6}A-1-RT-R	5'-GCCCACTTCTTGGCTCAGTG-3'
mouse *Egfr*-negative-m^{6}A-RT-F	5'-CCAACTTTTACCGAGCCCTG-3'
mouse *Egfr*-negative-m^{6}A-RT-R	5'-GACGGGCTGTTGAAGAAGCC-3'

mouse *Foxm1*-positive-m^{6}A-1-RT-F	5'-GAGCTGCAGATTCCCAGCCT-3'
mouse *Foxm1*-positive-m^{6}A-1-RT-R	5'-GATGTCTAGAAGGATCTTGCTGAGG -3'
mouse *Foxm1*-positive-m^{6}A-2-RT-F	5'-CTCAGCTGCCCTCAGGAAGA-3'
mouse *Foxm1*-positive-m^{6}A-2-RT-R	5'-GGGTCTCTAGAGAGCACAGACT-3'
mouse *Foxm1*-negative-m^{6}A-RT-F	5'-CCCTCGGGTTAGCTCATACC-3'
mouse *Foxm1*-negative-m^{6}A-RT-R	5'-ATAAGCGATGCTGCCAGAGG-3'
mouse *Hnf1a*-positive-m^{6}A-1-RT-F	5'-CCCTGTTCCTTAGGACCTGCAA-3'
mouse *Hnf1a*-positive-m^{6}A-1-RT-R	5'-GCAGGTCTCCCTCATAGCAGG-3'
mouse *Hnf1a*-negative-m^{6}A-RT-F	5'-AATGGCCTTGGAGAAACGCG-3'
mouse *Hnf1a*-negative-m^{6}A-RT-R	5'-CCTCCTCTGGGCTGAGGTT-3'
mouse *Hnf1β*-positive-m^{6}A-1-RT-F	5'-GAAACCCGTGGAGTCTCCCC-3'
mouse *Hnf1β*-positive-m^{6}A-1-RT-R	5'-GGCTCAGTTCATTAGCACACACC-3'
mouse *Hnf1β*-negative-m^{6}A-RT-F	5‘-ACAATCCCCAGCAATCTCAG-3’
mouse *Hnf1β*-negative-m^{6}A-RT-R	5‘-AGGCTGCTAGCCACACTGTT-3’
mouse *Hnf4a*-positive-m^{6}A-1-RT-F	5'-CCCTCAACTTCTTCACCCCT-3'
mouse *Hnf4a*-positive-m^{6}A-1-RT-R	5'-CGCTGGGAGAGAAGGGAGTT-3'
mouse *Hnf4a*-negative-m^{6}A-RT-F	5'-AGAGGTTCTGTCCCAGCAGA-3'
mouse *Hnf4a*-negative-m^{6}A-RT-R	5'-CACACGTCTGTGATGTTGGC-3'
mouse *Ldlr*-positive-m^{6}A-1-RT-F	5'-GAGGAGGAAGAGACCCACCC-3'
mouse *Ldlr*-positive-m^{6}A-1-RT-R	5'-GGGACTGGCTATGGTACCCA-3'
mouse *Ldlr*-negative-m^{6}A-RT-F	5'-GACGTGCTCCCAGGATGACT-3'
mouse *Ldlr*-negative-m^{6}A-RT-R	5'-TTTGGAGGCCGTGTCTCGG-3'
mouse *Onecut1*-positive-m^{6}A-1-RT-F	5'-TCATGCCCACCTGAATGCCC-3'
mouse *Onecut1*-positive-m^{6}A-1-RT-R	5'-CTTCCATTGCTGACCTGCGC-3'
mouse *Onecut1*-negative-m^{6}A-RT-F	5'-GAGCATGCCCACCACCTACA-3'
mouse *Onecut1*-negative-m^{6}A-RT-R	5'-GGTGGTGATGATGGTGAGGG-3'
mouse *Onecut2*-positive-m^{6}A-1-RT-F	5'-GCACCTACTTCTGCAACAAACAG-3'
mouse *Onecut2*-positive-m^{6}A-1-RT-R	5'-GCGTGCTTTTTCTGTTGATGGT-3'

mouse *Onecut2*-negative-m^{6}A-RT-F	5'-GTGGTTAATGTTGACCACATGGC-3'
mouse *Onecut2*-negative-m^{6}A-RT-R	5'-GTAAAAGTTAGCCCCGATTGGGT-3'
mouse *Ppara*-positive-m^{6}A-1-RT-F	5'-GCTTGGGGATGAAGAGGGCT-3'
mouse *Ppara*-positive-m^{6}A-1-RT-R	5'-CACCCCCATTTCGGTAGCAG-3'
mouse *Ppara*-positive-m^{6}A-2-RT-F	5'-ACTGGCCATCTTCTGACGTC-3'
mouse *Ppara*-positive-m^{6}A-2-RT-R	5'-CCCTCCATCCACTGGGCTAC-3'
mouse *Ppara*-negative-m^{6}A-RT-F	5'-ACACCCTCTCTCCAGCTTCC-3'
mouse *Ppara*-negative-m^{6}A-RT-R	5'-ACACTCGATGTTCAGGGCAC-3'
mouse *Sirt1*-positive-m^{6}A-1-RT-F	5'-GCTACAAGACAGGAATTGACAGATG-3'
mouse *Sirt1*-positive-m^{6}A-1-RT-R	5'-GTTCCCAATGCTGGTGGAGC-3'
mouse *Sirt1*-negative-m^{6}A-RT-F	5'-AGTTCCAGCCGTCTCTGTGT-3'
mouse *Sirt1*-negative-m^{6}A-RT-R	5'-GATCCTTTGGATTCCTGCAA-3'

Notes: m^{6}A-RIP-qPCR primers for m^{6}A positive regions were marked as "positive-m^{6}A-RT" (e.g., primers for two separate m^{6}A positive regions in *Ppara* were listed as *Ppara*-positive-m^{6}A-1-RT and *Ppara*-positive-m^{6}A-2-RT), and primers for m^{6}A negative regions were marked as "negative-m^{6}A-RT" (e.g., primers for m^{6}A negative regions in *Ppara* were listed as *Ppara*-negative-m^{6}A-RT).

Supplementary Table 2. Antibodies

**Antibodies**	**Source**	**Identifier**
m^{6}A (N6-methyladenosine) antibody	Synaptic Systems	Cat# 202003
anti-METTL3 antibody, Rabbit	Proteintech	Cat# 15073-1-AP
anti-METTL3 antibody, Rabbit mAb	Abcam	Cat# ab195352
anti-METTL14 antibody, Rabbit	Abclonal	Cat# A8530

mAb		
anti-GAPDH antibody, Rabbit mAb	Cell Signaling Technology	Cat# 2118
anti-β-actin antibody, Rabbit mAb	Cell Signaling Technology	Cat# 4970
mouse IgG	Beyotime	Cat# A7028
anti-Albumin antibody, Mouse mAb	R&D Systems	Cat# MAB1455
anti-SOX9 antibody, Rabbit	Sigma	Cat# AB5535
anti-Ki67 antibody	Abcam	Cat# AB15580
anti-alpha smooth muscle Actin (αSMA) antibody	Abcam	Cat# AB5694
anti-Cytokeratin 19 (CK19) antibody	Abcam	Cat# AB52625
anti-mouse IgG HRP-linked antibody	Cell Signaling Technology	Cat# 7076
anti-rabbit IgG HRP-linked antibody	Cell Signaling Technology	Cat# 7074
anti-Hnf4a antibody, Rabbit mAb	Cell Signaling Technology	Cat# 3113
anti-Hnf1a antibody	Abcam	Cat# AB11974
Recombinant Anti-PDGFR beta antibody	Abcam	Cat# AB32570
anti-IGF2BP1 antibody	Cell Signaling Technology	Cat# 8482
anti-Lamin B1 antibody	Cell Signaling Technology	Cat#13435

Supplementary Table 3. Reagents

**Reagents**	**Source**	**Identifier**
0.22 μm filter	PALL	PN4612
0.45 μm filter	PALL	PN4614
70 μm cell strainer	Sofra	251200
actinomycin D	Sigma-Aldrich	A1410
BODIPY™ 493/503	Invitrogen	D3922
ChamQ Universal SYBR qPCR Master Mix	Vazyme	Q711-03
ClonExpress II One Step Cloning kit	Vazyme	C112
Collagenase IV	Sigma-Aldrich	C5138
Complete Protease Inhibitor	Roche	04693132001
Dako Real™ Kit	Dako	K5007
Direct Mouse Genotyping kit	ApexBio Technology	k1025
DMEM/F-12 (1:1) basic medium	Thermo Scientific	C11330500BT
DMEM-high glucose medium	Thermo Scientific	C11995500BT
Dual-Luciferase Reporter Assay System	Promega	E1910
EDTA antigen retrieval buffer	ZSGB-BIO	ZLI-9072
Fetal bovine serum	PAN	P30-3302
GenElute™ mRNA Miniprep Kit	Sigma-Aldrich	MRN10
Glycogen	Beyotime	D0816
Hematoxylin	Baso	BA4041
Hoechst 33342	Beyotime	C1022
Hybond N+ membranes	GE Healthcare	RPN303B
Igepal CA-630	Sigma-Aldrich	I8896
Immonilon ECL Ultra Western HRP Substrate	Millipore	WBULS0500

In Situ Cell Death Detection Kit	Roche	11684795910
Lipofectamine™ 2000 kit	Invitrogen	11668-019
Olive Oil	MACKLIN	O815211
OCT compound	Servicebio	G6059
Penicillin/streptomycin	KeyGEN Biotech	KGY0023
Phanta Max DNA Polymerase	Vazyme	P505
Phosphatase inhibitor	Roche	04906831001
Propidium iodide solution	BD	556547
Polybrene	Sigma-Aldrich	H9268
PrimeScript™ RT Reagent Kit	Takara	RR047B
Protein A beads	Thermo Scientific	100-02D
Protein G beads	Thermo Scientific	100-04D
Proteinase K	Thermo Scientific	AM2546
Puromycin	Thermo Scientific	A1113803
RNasin® Ribonuclease Inhibitor-plus	Promega	N2611
Stbl3 *E. coli*	TransGen Biotech	CD521-01
Tamoxifen	Sigma-Aldrich	T5648
Triton™ X-100	Sigma-Aldrich	T8787
TRIzol	Ambion	15595018
Tween-20	Sigma-Aldrich	P1379
Type I Collagen, Rat tail	Invitrogen	A10483-01
Williams' Medium E	GIBCO	12551032
**Plasmids**		
pMIR-REPORT	Ambion	AM5795
pRL Renilla Luciferase Control Reporter Vector	Promega	E2231

Supplementary Datasets

Supplementary Dataset 1. Annotation of m^{6}A peaks.

Supplementary Dataset 1 was affiliated as an individual .xlsx file.

Supplementary Dataset 2. GO analysis of m^{6}A-modified genes.

Supplementary Dataset 2 was affiliated as an individual .xlsx file. Data were analyzed by over-representation test.

Supplementary Dataset 3. Time course liver tissue RNA-sequencing.

Supplementary Dataset 3 was affiliated as an individual .xlsx file. Differentially expressed genes were analyzed by nbinomWaldTest.

Supplementary Dataset 4. mRNA splicing analysis for genes in livers from different age of Control and cKO mice.

Supplementary Dataset 4 was affiliated as an individual .xlsx file. Data were analyzed by CASH [1].

Supplementary Dataset 5. RNA-sequencing of mouse primary hepatocyte treated with actinomycin D.

Supplementary Dataset 5 was affiliated as an individual .xlsx file.

Supplementary references

1 Wu, W. *et al.* CASH: a constructing comprehensive splice site method for detecting alternative splicing events. *Brief Bioinform* **19**, 905-917, doi:10.1093/bib/bbx034 (2018).
