# Supplementary material for: Mettl3-mediated mRNA m6A modification controls postnatal liver development by modulating the transcription factor Hnf4a
Source: Nat Commun. 2022 Aug 5;13:4555. doi: 10.1038/s41467-022-32169-4 (PMC9355946; doi:10.1038/s41467-022-32169-4)
Supplement: Supplementary file 2 — Reporting Summary [file 41467_2022_32169_MOESM2_ESM.pdf]

nature research

Corresponding author(s): Qi Zhang

Last updated by author(s): May 31, 2022

Reporting Summary

Nature Research wishes to improve the reproducibility of the work that we publish. This form provides structure for consistency and transparency in reporting. For further information on Nature Research policies, see our Editorial Policies and the Editorial Policy Checklist.

Statistics

For all statistical analyses, confirm that the following items are present in the figure legend, table legend, main text, or Methods section.

n/a	Confirmed	
☐	☒	The exact sample size (n) for each experimental group/condition, given as a discrete number and unit of measurement
☐	☒	A statement on whether measurements were taken from distinct samples or whether the same sample was measured repeatedly
☐	☒	The statistical test(s) used AND whether they are one- or two-sided<br>*Only common tests should be described solely by name; describe more complex techniques in the Methods section.*
☒	☐	A description of all covariates tested
☐	☒	A description of any assumptions or corrections, such as tests of normality and adjustment for multiple comparisons
☐	☒	A full description of the statistical parameters including central tendency (e.g. means) or other basic estimates (e.g. regression coefficient) AND variation (e.g. standard deviation) or associated estimates of uncertainty (e.g. confidence intervals)
☐	☒	For null hypothesis testing, the test statistic (e.g. F, t, r) with confidence intervals, effect sizes, degrees of freedom and P value noted<br>*Give P values as exact values whenever suitable.*
☒	☐	For Bayesian analysis, information on the choice of priors and Markov chain Monte Carlo settings
☒	☐	For hierarchical and complex designs, identification of the appropriate level for tests and full reporting of outcomes
☒	☐	Estimates of effect sizes (e.g. Cohen's d, Pearson's r), indicating how they were calculated

Our web collection on statistics for biologists contains articles on many of the points above.

Software and code

Policy information about availability of computer code

Data collection	RNA-seq and m6A-RIP-seq analysis: HISAT 2.1.0; exomePeak version 2.6.0 ; GSEA version 4.1.0; HOMER version 4.11; qPCR: LightCycler 480 Software 1.5.0; ZEN 2012; featureCounts version 2.0.1; STAR version 2.5.3a;
Data analysis	GraphPad Prism 8.0.2; ImageJ 1.8.0

For manuscripts utilizing custom algorithms or software that are central to the research but not yet described in published literature, software must be made available to editors and reviewers. We strongly encourage code deposition in a community repository (e.g. GitHub). See the Nature Research guidelines for submitting code & software for further information.

Data

Policy information about availability of data

All manuscripts must include a data availability statement. This statement should provide the following information, where applicable:

- Accession codes, unique identifiers, or web links for publicly available datasets
- A list of figures that have associated raw data
- A description of any restrictions on data availability

Data availability statement: Source data are provided with this paper. RNA-sequencing and m6A-RIP sequencing raw data and processed expression matrix are uploaded to GEO DataSets under accession code GSE197564. The sequencing reads were mapped to the mouse mm10 genome. All other data analyzed or generated in this study are provided along with the article.
Data in these Figures and Supplementary Figures (Figure 5a-5d, Figure 6a, SFigure 5b-5f, SFigure 6a-6b, SFigure 6g, SFigure 7a, and SFigure 7g-7i) have been associated with the raw data mentioned above.
There are not any restrictions on data availability.

Field-specific reporting

Please select the one below that is the best fit for your research. If you are not sure, read the appropriate sections before making your selection.

- [x] Life sciences
- [ ] Behavioural & social sciences
- [ ] Ecological, evolutionary & environmental sciences

For a reference copy of the document with all sections, see nature.com/documents/nr-reporting-summary-flat.pdf

Life sciences study design

All studies must disclose on these points even when the disclosure is negative.

Sample size

The sample size for each experiment was described in Figure Legends. Sample size in mouse experiments was determined to get biological meaningful results, and thus enough to reflect the huge differences between Control and Mettl3-cKO mice. Detailed mice numbers in each experiment were determined by the number of mice born in the cages designed for indicated experiments. Sample sizes for qPCR, western blot, dual-luciferase report assay, and other cell and molecular experiments were designed for at least three samples in each group for analysis.

Data exclusions

No data were excluded from the analysis.

Replication

All the cell culture experiments were repeated at least three times, and we made sure that cells in these data were cultured strictly sticking on sterile and mycoplasma-free conditions. For animal studies, more than three mice in each group were used in animal experiments, which was sufficient to get credible conclusions. Considering the animal welfare, especially for Mettl3-cKO mice which will die within 7 weeks old, we try to get supportive conclusions with the fewest possible replicate experiments. Two individuals were used in each group in RNA-seq and m6A-RIP-seq at each time point, which is enough to get biologically meaningful differences, particularly considering the tremendous phenotype differences between Control and Mettl3-cKO mice. Also, potential targets identified in the RNA-seq were further validated with RT-qPCR. Two replicates were used for LC-MS/MS to detect bulk mRNA m6A levels in each group, reflecting the m6A decreased situation in Mettl3-cKO and icKO mice compared to Control. Western Blot was confirmed by at least four independent experiments with similar results and thus believed to be reliable.

Randomization

Mice with different genotypes were randomly divided into different groups. For example, when Control or cKO mice were divided into two groups, each individual was chosen just randomly and allocated to different groups. Likewise, cultured cells in different dishes were randomly allocated into different treatment groups.

Blinding

H&E staining, BODIPY staining, Masson staining, IHC staining, and mouse experiments in this article were performed and analyzed by investigators who were blinded for experimental designs. Blinding was not performed for cell-related experiments because the investigator must know the treatment for each group. For western blot, qPCR, MERIP-qPCR, genotyping, and dual-luciferase report assay experiments, the investigator was not blinded since these experiments were conducted by the same person.

Reporting for specific materials, systems and methods

We require information from authors about some types of materials, experimental systems and methods used in many studies. Here, indicate whether each material, system or method listed is relevant to your study. If you are not sure if a list item applies to your research, read the appropriate section before selecting a response.

Materials & experimental systems

n/a	Involved in the study
[ ]	[x] Antibodies
[ ]	[x] Eukaryotic cell lines
[x]	[ ] Palaeontology and archaeology
[ ]	[x] Animals and other organisms
[ ]	[x] Human research participants
[x]	[ ] Clinical data
[x]	[ ] Dual use research of concern

Methods

n/a	Involved in the study
[x]	[ ] ChIP-seq
[x]	[ ] Flow cytometry
[x]	[ ] MRI-based neuroimaging

Antibodies

Antibodies used

1. m6A (N6-methyladenosine) antibody, Synaptic Systems Cat# 202003. 1 ug for each reaction in m6A-RIP-qpcr experiment
2. anti-METTL3 antibody, Rabbit Proteintech Cat# 15073-1-AP. 1:1000 for western blot experiment
3. anti-METTL3 antibody, Rabbit mAb Abcam Cat# ab195352. 1:200 for IHC experiment
4. anti-METTL14 antibody, Rabbit mAb Abclonal Cat# A8530. 1:1000 for western blot experiment
5. anti-GAPDH antibody, Rabbit mAb Cell Signaling Technology Cat# 2118. 1:4000 for western blot experiment
6. anti-β-actin antibody, Rabbit mAb Cell Signaling Technology Cat# 4970. 1:1000 for western blot experiment
7. mouse IgG Beyotime Cat# A7028. 1 ug for each reaction in m6A-RIP-qpcr experiment
8. anti-Albumin antibody, Mouse mAb R&D Systems Cat# MAB1455 (Clone # 188835). 1:1000 for western blot experiment

9. anti-SOX9 antibody, Rabbit Sigma Cat# AB5535. 1:1000 for western blot experiment. 1:100 for IHC experiment
10. anti-Ki67 antibody Abcam Cat# AB15580. 1:1000 for western blot experiment. 1:500 for IHC experiment
11. anti-alpha smooth muscle Actin (αSMA) antibody Abcam Cat# AB5694. 1:1000 for western blot experiment. 1:500 for IHC experiment
12. anti-Cytokeratin 19 (CK19) antibody Abcam Cat# AB52625. 1:200 for IHC experiment
13. anti-mouse IgG HRP-linked antibody Cell Signaling Technology Cat# 7076. 1:2000 for western blot experiment
14. anti-rabbit IgG HRP-linked antibody Cell Signaling Technology Cat# 7074. 1:2000 for western blot experiment
15. anti-Hnf4α antibody, Rabbit mAb Cell Signaling Technology Cat# 3113. 1:1000 for western blot experiment
16. anti-Hnf1α antibody Abcam Cat# AB11974. 1:1000 for western blot experiment
17. Recombinant Anti-PDGFR beta antibody Abcam Cat# AB32570. 1:1000 for western blot experiment
18. anti-IGF2BP1 (IMP1) antibody, Rabbit mAb Cell Signaling Technology Cat#8482. 1:1000 for western blot experiment. 1 ug for each reaction in RIP experiment

Validation

All of these antibodies are commercially available and have been test to recognize for both human and mouse species. The quality control and validation are provided by the manufacturer's websites:
1. m6A (N6-methyladenosine) antibody, Synaptic Systems Cat# 202003.(https://www.sysy.com/product/202003#list)
2. anti-METTL3 antibody, Rabbit Proteintech Cat# 15073-1-AP.(https://www.ptgcn.com/products/METTL3-Antibody-15073-1-AP.htm)
3. anti-METTL3 antibody, Rabbit mAb Abcam Cat# ab195352. (https://www.abcam.cn/mettl3-antibody-epr18810-ab195352.html)
4. anti-METTL14 antibody, Rabbit mAb Abclonal Cat# A8530. (https://abclonal.com.cn/catalog/A8530)
5. anti-GAPDH antibody, Rabbit mAb Cell Signaling Technology Cat# 2118. (https://www.cellsignal.cn/products/primary-antibodies/gapdh-14c10-rabbit-mab/2118?site-search-type=Products&N=4294956287&Ntt=2118&fromPage=plp&_requestid=563069)
6. anti-β-actin antibody, Rabbit mAb Cell Signaling Technology Cat# 4970.(https://www.cellsignal.cn/products/primary-antibodies/b-actin-13e5-rabbit-mab/4970?site-search-type=Products&N=4294956287&Ntt=4970&fromPage=plp&_requestid=563140)
7. mouse IgG Beyotime Cat# A7028. (https://www.beyotime.com/product/A7028.htm)
8. anti-Albumin antibody, Mouse mAb R&D Systems Cat# MAB1455 (Clone # 188835).(https://www.rndsystems.com/cn/products/human-serum-albumin-antibody-188835_mab1455)
9. anti-SOX9 antibody, Rabbit Sigma Cat# AB5535.(https://www.sigmaaldrich.cn/CN/zh/product/mm/ab5535af488)
10. anti-Ki67 antibody Abcam Cat# AB15580. (https://www.abcam.cn/ki67-antibody-ab15580.html)
11. anti-alpha smooth muscle Actin (αSMA) antibody Abcam Cat# AB5694. (https://www.abcam.cn/alpha-smooth-muscle-actin-antibody-ab5694.html)
12. anti-Cytokeratin 19 (CK19) antibody Abcam Cat# AB52625. (https://www.abcam.cn/cytokeratin-19-antibody-ep1580y-cytoskeleton-marker-ab52625.html)
13. anti-mouse IgG HRP-linked antibody Cell Signaling Technology Cat# 7076. (https://www.cellsignal.cn/products/secondary-antibodies/anti-mouse-igg-hrp-linked-antibody/7076?site-search-type=Products&N=4294956287&Ntt=7076&fromPage=plp&_requestid=563191)
14. anti-rabbit IgG HRP-linked antibody Cell Signaling Technology Cat# 7074. (https://www.cellsignal.cn/products/secondary-antibodies/anti-rabbit-igg-hrp-linked-antibody/7074?site-search-type=Products&N=4294956287&Ntt=7074&fromPage=plp&_requestid=563236)
15. anti-Hnf4α antibody, Rabbit mAb Cell Signaling Technology Cat# 3113. (https://www.cellsignal.cn/products/primary-antibodies/hnf4a-c11f12-rabbit-mab/3113?site-search-type=Products&N=4294956287&Ntt=3113&fromPage=plp&_requestid=563300)
16. anti-Hnf1α antibody Abcam Cat# AB11974. (https://www.abcam.cn/hnf1-alpha-antibody-ab11974.html)
17. Recombinant Anti-PDGFR beta antibody Abcam Cat# AB32570. (https://www.abcam.cn/pdgfr-alpha--pdgfr-beta-antibody-y92-c-terminal-ab32570.html)
18. anti-IGF2BP1 (IMP1) antibody, Rabbit mAb Cell Signaling Technology Cat#8482. (https://www.cellsignal.cn/products/primary-antibodies/imp1-d33a2-rabbit-mab/8482?site-search-type=Products&N=4294956287&Ntt=8482&fromPage=plp&_requestid=563340)

Eukaryotic cell lines

Policy information about cell lines

Cell line source(s)

HEK293T and HepG2 were obtained from ATCC.

Authentication

The cell line has not been authenticated recently.

Mycoplasma contamination

All cell lines were tested negative for mycoplasma contamination.

Commonly misidentified lines
(See ICLAC register)

No ICLAC lines were used in this study

Animals and other organisms

Policy information about studies involving animals; ARRIVE guidelines recommended for reporting animal research

Laboratory animals

All the mice were C57BL/6J background and housed in a specific pathogen-free facility under 12 h light/dark cycle, temperature of 24 ±2°C, humidity between 30-70%, with access to food and water ad libitum. Both C57BL/6J male and female mice for postnatal liver

	development experiments were studied between 0-8 weeks. C57BL/6J male mice for adult liver homeostasis experiments were studied between 4-20 weeks.
Wild animals	No wild animals were involved in this study.
Field-collected samples	No field-collected samples were involved in this study.
Ethics oversight	All Mice were used in accordance with the Guide for Care and Use of Laboratory Animals of the National Institute of Health, and protocols were approved by the Institutional Animal Care and Use Committee of The Third Affiliated Hospital of Sun Yat-sen University.

Note that full information on the approval of the study protocol must also be provided in the manuscript.

Human research participants

Policy information about studies involving human research participants

Population characteristics	Human liver specimens were obtained from clinical patients of different ages to test METTL3 and METTL14 hepatic protein levels and show patterns of these two proteins. All liver specimens used in this study showed no liver disease and were thus considered as normal liver samples.
Recruitment	Human liver tissues were obtained from donation after cardiac death during liver transplantation in the Third Affiliated Hospital of Sun Yat-sen University.
Ethics oversight	Informed consent was obtained from all individuals and recorded in the electronic database. The study has been approved by the Medical Ethical Committees of the Third Affiliated Hospital of Sun Yat-sen University. The study design and conduct complied with all relevant regulations regarding the use of human study participants and was conducted following the criteria set by the Declaration of Helsinki.

Note that full information on the approval of the study protocol must also be provided in the manuscript.
