# Supplementary material for: Mettl3-mediated mRNA m6A modification controls postnatal liver development by modulating the transcription factor Hnf4a
Source: Nat Commun. 2022 Aug 5;13:4555. doi: 10.1038/s41467-022-32169-4 (PMC9355946; doi:10.1038/s41467-022-32169-4)
Supplement: Supplementary file 3 — Description of Additional Supplementary Files [file 41467_2022_32169_MOESM3_ESM.pdf]

Title: Supplementary Dataset 1.

Description: Annotation of m6A peaks

Title: Supplementary Dataset 2.

Description: GO analysis of m6A-modified genes

Title: Supplementary Dataset 3.

Description: Time course liver tissue RNA-sequencing

Title: Supplementary Dataset 4.

Description: mRNA splicing analysis for genes in livers from different age of Control and cKO mice

Title: Supplementary Dataset 5.

Description: RNA-sequencing of mouse primary hepatocyte treated with actinomycin D
